# The nature of human sperm head vacuoles: a systematic literature review

**DOI:** 10.1186/2051-4190-23-3

**Published:** 2013-08-29

**Authors:** Florence Boitrelle, Bruno Guthauser, Laura Alter, Marc Bailly, Robert Wainer, François Vialard, Martine Albert, Jacqueline Selva

**Affiliations:** Department of Reproductive Biology, Cytogenetics and Gynaecology, Poissy General Hospital, F-78303 Poissy, France; EA 2493, Versailles University of Medicine and Science, F-78000 Versailles, France

**Keywords:** Human sperm vacuoles, MSOME, IMSI, High-magnification, Nuclear, Chromatin, Vacuole spermatique, MSOME, IMSI, Nature nucléaire, Comdensation, Chromatine

## Abstract

Motile sperm organelle morphology examination (MSOME) involves the use of differential interference contrast microscopy (also called Nomarski contrast) at high magnification (at least 6300x) to improve the observation of live human spermatozoa. In fact, this technique evidences sperm head vacuoles that are not necessarily seen at lower magnifications - particularly if the vacuoles are small (i.e. occupying <4% of the sperm head’s area). However, a decade after MSOME's introduction, it is still not clear whether sperm head vacuoles are nuclear, acrosomal and/or membrane-related in nature. In an attempt to clarify this debate, we performed a systematic literature review in accordance with the PRISMA guidelines. The PubMed database was searched from 2001 onwards with the terms "MSOME", “human sperm vacuoles”, "high-magnification, sperm”. Out of 180 search results, 21 relevant English-language publications on the nature of human sperm head vacuoles were finally selected and reviewed. Our review of the literature prompted us to conclude that sperm-head vacuoles are nuclear in nature and are related to chromatin condensation failure and (in some cases) sperm DNA damage.

## Résumé

Le MSOME (motile sperm organelle morphology examination) est une technique d’observation des spermatozoïdes mobiles à fort grossissement (>6300x) à l’aide du contraste interférentiel différentiel de Nomarski. Avec cette technique, des anomalies de la tête spermatique comme les vacuoles peuvent être observées alors qu’elles demeurent souvent invisibles à plus faible grossissement, notamment quand elles sont petites et qu’elles occupent moins de 4% de la surface de la tête. Depuis l’introduction du MSOME dans les années 2000, plusieurs études se sont intéressées à la nature des vacuoles. Sont-elles de nature nucléaire ? de nature acrosomique ? de nature membranaire ? Pour répondre à ces questions, nous avons réalisé une revue de la littérature en suivant les règles PRISMA. Les études publiées sur le sujet entre 2001 et aujourd’hui ont été recherchées dans la base Pubmed en utilisant les mots clés : "MSOME", “human sperm vacuoles” et "high-magnification, sperm”. Parmi les 180 études retrouvées, 21 publications écrites en langue Anglaise et traitant de la nature des vacuoles spermatiques ont été sélectionnées et étudiées. Au total, cette revue de la littérature conclut que les vacuoles sont de nature nucléaire, en lien avec une moindre condensation de la chromatine spermatique. Cette moindre condensation chromatinienne représentant un facteur de susceptibilité aux dommages de l’ADN (fragmentation, dénaturation par exemple), les spermatozoïdes vacuolés peuvent aussi présenter plus de dommages de l’ADN que les spermatozoïdes sans vacuole.

## Introduction

Since the first use of intracytoplasmic sperm injection (ICSI) in the early 1990s [1], this technique has become a powerful tool for infertile couples - particularly in cases of severe male infertility and low sperm counts. In ICSI, the “best-looking” or the “morphologically most normal” live spermatozoon is chosen. However, it is well known that selection of a normal spermatozoon during ICSI does not guarantee nuclear quality or enable the detection of nuclear defects [2, 3]. Furthermore, the acrosomal status of spermatozoa selected under ICSI magnification (i.e. acrosome-reacted or not) is not known. Given that (i) nuclear defects are known to influence early and late embryo development (for a review, see [4]) and (ii) injection of the acrosome into the oocyte during ICSI may harm embryo development [5], some researchers have tried to improve the quality of spermatozoon observation by establishing correlations between the morphology of a viable (and subsequently injectable) spermatozoon and its inherent nuclear or acrosomal qualities.

Hence, motile sperm organelle morphology examination (MSOME, using Nomarski differential interferential contrast microscopy and high magnification, >6300×) was developed in the early 2000s [6]. This sperm observation technique reportedly enables better assessment of a spermatozoon’s morphology and better visualization of sperm head vacuoles – structures that are not visible (particularly if they are small, i.e. occupying <4% of the sperm head’s area) at a conventional ICSI-like magnification [6]. Sperm head vacuoles can be classified by size (large, small), position (anterior, posterior), deepness (deep-lying or superficial) and number (single or multiple). They are found in semen with normal characteristics as well as in semen with abnormal characteristics. Vacuole size (relative to sperm head size) was recently found to be negatively correlated with poor sperm morphology [7]. In terms of the links between the presence of vacuoles and embryo development, it was recently shown that individual spermatozoa differ in their ability to produce an embryo capable of implanting. In fact, two different studies showed that the injection of morphometrically normal spermatozoa with no vacuoles or only one small vacuole was associated with significantly higher blastocyst [8] and/or pregnancy rates [8, 9] (relative to the injection of morphometrically abnormal spermatozoa or morphometrically normal spermatozoa with two or more small vacuoles or one large vacuole). In a similar approach, Cassuto et al. [10] established a score that took account of sperm head morphology, base morphology and the number of vacuoles. The researchers reported that pregnancy rate varied as a function of the score, with lower rates obtained for abnormal spermatozoa with vacuoles. Although one group has reported that the injection of vacuole-free spermatozoa was associated with lower blastocyst rates, the cohort was very small and, one third of the patients presented azoospermia [11]. To summarize, it seems that the presence of one or more vacuoles can influence blastocyst and pregnancy rates. Why might this be? And are vacuoles nuclear, acrosomal, and/or membrane-related in nature? Do large vacuoles differ from small vacuoles, other than in size? Several recent studies have been performed with a view to answering these questions. The objective of the present literature review was to assess the nature of human sperm head vacuoles.

## Methods

We performed a systematic review of the relevant literature, in accordance with the PRISMA guidelines. We searched the PubMed database (between January 2001 and March 2013) with the following search terms: "MSOME", "human sperm vacuoles" and "high-magnification, sperm, vacuole". The publications’ titles, abstracts and reference lists were screened. Only relevant publications in English were selected and reviewed. A study was considered to be relevant if it assessed the nature of human sperm head vacuoles in terms of morphological or/and functional criteria. We examined, compared and discussed study methodologies and results. We divided our results into two sections, covering the structural and then functional aspects of human sperm head vacuoles.

## Results and discussion

Our PubMed search identified a total of 180 publications (29 using the term "MSOME", 129 using the term "human sperm vacuole" and 22 using the term "high-magnification, sperm") indexed between January 2001 and March 2013. After screening the titles, abstracts and reference lists, we included 21 English-language publications in our review.

### Structural aspects

Human sperm vacuoles were first described as “nuclear holes” when examined by electron microscopy and 2-D imaging [12]. Thanks to higher-resolution techniques and technical progress in microscopic imaging, it was recently showed that vacuoles were nuclear concavities and not nuclear holes [13–15].

Atomic force microscopy is a very-high-resolution type of scanning microscopy, which provides nanometre-scale resolution. Using this technique, large vacuoles (occupying more than 25% of the sperm head area) have been described as thin nuclear areas where the plasma membrane was intact but sunken [15] or where the sperm plasma membrane fell [13]. The vacuoles were then described as “hollows” or “concavities”. Using three-dimensional (3D) deconvolution microscopy (a technique with a resolution of about 100 nm) large and small vacuoles (<4% of the head area) were described as thumbprint-like and pocket-like nuclear concavities, respectively [14, 15]. Furthermore, vacuoles that appeared to be small when viewed at the surface were sometimes larger and deeper than expected [14]. In summary, structural studies have described vacuoles as nuclear concavities.

### Functional aspects

Various assays are used to explore sperm nuclear quality (for a review, see [16, 17]). In sperm chromatin condensation during spermiogenesis, histones are replaced by protamines. The two most frequently used chromatin condensation assays were chromomycin A3 (CMA3) staining (based on *in situ* competition with protamines) and aniline blue (AB) staining (based on the detection of residual histones in the sperm head). To assess sperm DNA damage and fragmentation, the acridine orange (AO) assay is used to evidence single-strand DNA breaks and the terminal deoxynucleotidyl transferase-mediated dUTP-nick end-labelling (TUNEL) assay is used to evidence both single- and double-strand DNA breaks. Lastly, sperm fluorescence *in situ* hybridization (FISH) is used to assess sperm chromosome content.

#### Links between vacuoles and sperm chromatin condensation status

The relationship between the presence of vacuoles and the degree of sperm chromatin condensation has been studied extensively (for an overview, see Table 1). Some researchers have focused on chromatin condensation in spermatozoa with large vacuoles. Below, we deliberately mention whether the studies in question assessed the chromatin condensation of spermatozoa with large vacuoles (regardless of the potential presence of other sperm abnormalities) or with a single, large vacuole as the only abnormality (i.e. in otherwise morphologically normal sperm). In Cassuto et al.’s study of 26 patients, spermatozoa with an abnormally shaped head (i.e. an abnormal base and/or an asymmetric nuclear extrusion) and at least one large vacuole (although the size was not specified) were selected under high magnification [18]. This type of spermatozoa was referred to as “score-0”. The researchers compared the degree of chromatin condensation (according to AB staining) of score-0 spermatozoa with that of unselected spermatozoa (i.e. those present in the sperm after two-layer density centrifugation). The proportion of spermatozoa with chromatin condensation failure was higher for score-0 spermatozoa than for unselected spermatozoa (19.5% vs. 10.1%, respectively; p<0.0001). Perdrix et al. studied 20 patients and selected spermatozoa with one large vacuole (occupying >13% of the sperm head area), regardless of whether or not the latter presented other morphological abnormalities [19]. The proportion of spermatozoa with chromatin condensation failure (according to AB staining) was higher for spermatozoa with large vacuoles than for spermatozoa from whole sperm (50.4% vs. 26.5%, respectively; p<0.0001). Franco et al. [20] studied 66 patients and selected spermatozoa with one or more large vacuole (occupying ≥ 50% of the sperm head area) – again, regardless of whether or not these spermatozoa presented other morphological abnormalities. The researchers found that the spermatozoa with large vacuoles were more likely to present abnormal chromatin packaging (as assessed by the CMA3 assay) than morphologically normal, vacuole-free spermatozoa were (53.2% vs. 40.3%, respectively; p<0.0001). Interestingly, some researchers have assessed the chromatin condensation status of spermatozoa in which the presence of a large vacuole was the only abnormality [15]. In a study of 15 patients, morphologically normal spermatozoa with one large vacuole (>25% of the head area) were more likely to present chromatin condensation failure (as assessed by AB staining) than vacuole-free, morphologically normal spermatozoa (36.2% vs. 7.6%, respectively; p<0.0001). Although these studies all found an association between the presence of one or more large vacuoles and chromatin condensation failure, few have studied the nature of small vacuoles. Only one group recently reported that chromatin condensation failure (as assessed by AB staining) was also related to the nature of small vacuoles [14]. Spermatozoa with more than two small vacuoles (each occupying less than 4% of the sperm head area) but that were otherwise normal (i.e. free of morphological abnormalities) were indeed more likely to have non-condensed chromatin than morphologically normal spermatozoa without vacuoles were (39.8% vs. 9.3%, respectively; p<0.0001). In summary, several concordant studies have established links between the presence of vacuoles and chromatin condensation failure.

**Table 1:** **Links between vacuoles and sperm chromatin condensation status**

	Number of patients	Chromatin condensation assessment	Vacuolated spermatozoa			Spermatozoa used as “controls”		P
			Number and size of vacuoles	Presence of other potential abnormalities	Proportion of vacuolated spermatozoa with a non condensed chromatin (%)	Type of spermatozoa used as “controls”	Proportion of “control” spermatozoa with a non condensed chromatin (%)	
Cassuto 2012 [18]	26	AB	At least one vacuole (size not specified)	Yes	19.5	Unselected spermatozoa (obtained after two-layer density centrifugation)	10.1	**p<0.0001**
Perdrix 2011 [19]	20	AB	A single vacuole occupying > 13% of the sperm head area	Yes	50.4	Whole sperm	26.5	**p<0.0001**
Franco 2012 [20]	66	CMA3	At least one vacuole occupying > 50% of the sperm head area	Yes	53.2	Morphologically normal and vacuole-free	40.3	**p<0.0001**
Boitrelle 2011 [16]	15	AB	A single vacuole occupying > 25% of the sperm head area	No	36.2	Morphologically normal and vacuole-free	7.6	**p<0.0001**
Boitrelle In press	15	AB	At least three vacuoles occupying each < 4% of the sperm head area	No	39.8	Morphologically normal and vacuole-free	9.3	**p<0.0001**

Studies (with sample sizes and methodological details) evaluating the relationship between the presence of vacuoles (or not) and sperm chromatin condensation status. *AB*: aniline blue staining, *CMA3*: chromomycin A3 staining. P values in bold type are statistically significant.

#### The relationship between the presence of vacuoles and DNA fragmentation/damage

Since the degree of chromatin condensation confers susceptibility to nuclear DNA damage during the spermatozoon's journey though the male genital tract [17, 21], several research groups have studied the putative links between the presence of sperm head vacuoles and DNA damage. A positive strong correlation (Spearman’s coefficient r=0.73; p=0.01) between the proportion of spermatozoa with vacuoles (regardless of the vacuole's size and the presence or absence of morphological sperm abnormalities) and the proportion of spermatozoa with denatured DNA (according to an AO assay) was reported for 10 patients with severe teratozoospermia (<5% of normal sperm forms, according to Kruger’s criteria) [22]. In a study of 50 patients, positive but weak correlations between the presence of vacuoles (regardless of the presence or absence of morphometric sperm abnormalities) and the proportion of spermatozoa with DNA fragmentation (in a TUNEL assay) was reported (Spearman’s coefficient for large vacuoles and for small vacuoles; r=0.3; p=0.03) [23]. Although other researchers have found a positive but weak correlation between the proportion of spermatozoa with large vacuoles (> 50% of the sperm head area) and the proportion of spermatozoa with DNA fragmentation (in a TUNEL assay) (Spearman’s coefficient r=0.1; p<0.05), there was no correlation between DNA fragmentation and the presence of small vacuoles (Spearman’s coefficient r=−0.05) [24]. So, there may be a correlation between the presence of large vacuoles and DNA damage. However, assessment of DNA fragmentation or damage in individually selected spermatozoa is more reliable basis for determining putative links between DNA damage and the presence of vacuoles (for an overview, see Table 2).

**Table 2:** **The relationship between the presence of vacuoles and DNA fragmentation/damage**

	Number of patients	DNA damages assessment	Vacuolated spermatozoa			Spermatozoa used as “controls”		P
			Number and size of vacuoles	Presence of other potential abnormalities	Proportion of vacuolated spermatozoa with DNA damages (%)	Type of spermatozoa used as “controls”	Proportion of “control” spermatozoa with DNA damages (%)	
Franco 2008 [26]	30	AO TUNEL	At least one vacuole occupying > 50% of the sperm head area	Yes	DNA denaturation: 67.9 DNA fragmentation: 29.1	Morphologically normal and vacuole-free	DNA denaturation: 33.1 DNA fragmentation: 15.9	**P<0.0001 P<0.0001**
Garolla 2008 [25]	10	AO TUNEL	At least one vacuole (size not specified)	No	DNA denaturation: 71.9 DNA fragmentation: 40.1	Morphologically normal and vacuole-free	DNA denaturation: 5.3 DNA fragmentation: 9.3	**P<0.001 P<0.001**
Wilding 2011 [27]	5	TUNEL	Multiple vacuoles occupying each > 4% of the sperm head area	No	14.4	Morphologically normal with no more than one small vacuole	4.2	**P=0.03**
Hammoud 2013 [28]	8	TUNEL	Multiple vacuoles (size not specified)	No	15.9 ^a^ (for anterior vacuoles) 22.5 ^b^ (for posterior ones)	Morphologically normal and vacuole-free	4.1^a,b^	**a P<0.05 b P<0.001**
Cassuto 2012 [18]	26	TUNEL	At least one vacuole (size not specified)	Yes	4.2	Unselected spermatozoa (obtained after two-layer density centrifugation)	3.7	NS
Perdrix 2011 [19]	20	TUNEL	A single vacuole occupying > 13% of the sperm head area	Yes	1.7	Whole sperm	8.6	NS
Boitrelle 2011 [16]	15	TUNEL	A single vacuole occupying > 25% of the sperm head area	No	1.3	Morphologically normal and vacuole-free	0.7	NS
Watanabe 2011 [30]	20	TUNEL	A single vacuole with a diameter > 1.5 μm	No	2.3	Morphologically normal and vacuole-free	0.0	NS

Studies (with sample sizes and methodological details) evaluating the relationship between the presence of vacuoles (or not) and DNA damages. *AO*: acridine orange test, *TUNEL*: the terminal deoxynucleotidyl transferase-mediated dUTP-nick end-labelling. P values in bold type are statistically significant. a: P obtained when proportion of ‘control’ spermatozoa DNA fragmentation rates were compared with DNA fragmentation rates of spermatozoa with anterior vacuoles. b: P obtained when proportion of ‘control’ spermatozoa DNA fragmentation rates were compared with DNA fragmentation rates of spermatozoa with posterior vacuoles.

Individually selected spermatozoa with large vacuoles [25, 26] or several large or small vacuoles [27, 28] have been found to present high levels of DNA damage. In a study of 30 patients, spermatozoa with large vacuoles (>50% of the sperm head area) and potentially other abnormalities had significantly higher levels of DNA denaturation (according to an AO assay) and DNA fragmentation (according to a TUNEL assay) than vacuole-free, morphologically normal spermatozoa (67.9% vs. 33.1%, respectively, for DNA denaturation; 29.1% vs. 15.9%, respectively, for DNA fragmentation; both p<0.0001) [26]. However, the vacuolated sperm head's shape was not described in the latter study. This would probably have generated interesting data, since it was recently reported that spermatozoa with an abnormal shape (as assessed by MSOME) were more likely to have fragmented DNA (according to a TUNEL assay) than spermatozoa with a normal shape were [29]. In a study of 10 patients, Garolla et al. [25] showed that otherwise normal spermatozoa with at least one large vacuole (without specifying their size) had significantly higher levels of DNA denaturation (as assessed by the AO assay) and DNA fragmentation (as assessed by a TUNEL assay) than vacuole-free, morphometrically normal spermatozoa (71.9% vs. 5.3%, respectively, for DNA denaturation; 40.1% vs. 9.3% respectively, for DNA fragmentation; both p<0.001). Furthermore, Wilding et al. found that otherwise normal spermatozoa with several vacuoles (>4% of the head area) had significantly higher levels of DNA fragmentation (according to a TUNEL assay) than normal spermatozoa with no more than one small vacuole did (14.4% vs. 4.2%, respectively; p=0.03) [27]. Likewise, Hammoud et al. [28] reported that morphometrically normal spermatozoa with several anterior or posterior vacuoles (size not specified) had significantly higher levels of DNA fragmentation (in a TUNEL assay) than morphometrically normal, vacuole-free spermatozoa did (15.9% for anterior vacuoles and 22.5% for posterior vacuoles vs. 4.1% for vacuole-free spermatozoa, p<0.05 and p<0.001 for anterior and posterior vacuoles, respectively). Interestingly, the normal spermatozoa selected under ICSI-like magnification (×200) also had higher levels of DNA fragmentation than normal, vacuole-free spermatozoa selected with MSOME did (18.7% vs. 4.1% respectively; p<0.001).

In contrast, other researchers have failed to observe a link between DNA damage and individually selected spermatozoa with large vacuoles [15, 18, 19, 30]. In a study of 26 patients, Cassuto et al. did not observe a significant difference in the DNA fragmentation rate (according to a TUNEL assay) when comparing "score-0" spermatozoa (those with an abnormally shaped head and at least one large vacuole) and spermatozoa in the migrated sperm fraction (4.2% vs. 3.7%, respectively) [18]. Significantly lower sperm DNA fragmentation rates were even observed in spermatozoa with large vacuoles (>13% of the head area) and potentially other abnormalities, when compared with unselected spermatozoa from whole sperm (1.7% vs. 8.6%, p<0.0001) [19]. Interestingly, two studies (with 15 and 20 patients, respectively) did not observe a significant difference in DNA fragmentation rate (as assessed by a TUNEL assay) between morphometrically normal spermatozoa with one large vacuole (>25% of the head surface area in one study [15] and a diameter >1.5 μm in the other [30]) and those lacking vacuoles (respectively 1.3% vs. 0.7% in one study [15] and 2.3% vs. 0.0% in the other ) [15, 30]. It should be noted that in the four latter studies, the spermatozoon DNA fragmentation rates in whole semen were low.

In summary, there are divergent data on the putative link between human sperm vacuoles and DNA damage. It has been suggested that these discrepancies are due to differences in the extent of chromatin condensation failure (and thus susceptibility to DNA damage) from one patient to another [26, 28]. In fact, there is some evidence to suggest that DNA is more susceptible to damage when histones are not replaced by protamines during spermiogenesis [31, 32]. Hence, vacuoles might be associated with chromatin condensation failure and DNA damage only in patients with high overall DNA damage rates in semen and not in patients with low DNA damage rates in semen. Further research is needed to define the circumstances under which spermatozoa with vacuoles and non-condensed chromatin are more likely to present DNA damage.

#### Relationships between vacuoles and sperm chromosome content

Lastly, some researchers have focused on the putative links between the presence of vacuoles and the sperm chromosome content. In a study of 50 patients, de Almeida Ferreira Braga et al. failed to observe any correlation between the presence of vacuoles (whether large or small) and sperm aneuploidy (according to FISH with probes for chromosomes X, Y, 13, 18 and 21) (Spearman’s coefficient r=0.09 and r=0.03 for large and small vacuoles, respectively) [23]. However, the assessment of chromosome content in individually selected spermatozoa is a more robust way of investigating potential links between the presence of vacuoles and potential sperm aneuploidies.

In two studies of patients with a normal karyotype, the presence of a large vacuole was shown to be associated with abnormal sperm chromosomal status, as assessed by sperm FISH [19, 25]. In a study of 10 patients, Garolla et al. [25] showed that spermatozoa with at least one large vacuole (size not specified) but that were otherwise morphologically normal were more likely to be aneuploid (using FISH probes for chromosomes X, Y and 18) than vacuole-free morphometrically normal spermatozoa (5.1% vs. 0.0%, respectively, p not stated). Furthermore, in a study of 20 patients, total chromosome abnormalities (as assessed with FISH probes for chromosomes 18, 24, X and Y) were significantly more common in spermatozoa with a single, large vacuole (>13% of the sperm head area) and potentially other abnormalities than in spermatozoa from whole sperm (7.8% vs. 1.3%, respectively; p<0.0001) [19]. The researchers suggested later that these abnormalities were due to architectural disorganization of the chromatin in spermatozoa with large vacuoles [33].

In contrast, other researchers have failed to observe an elevated risk of chromosomal abnormalities in spermatozoa with large vacuoles [15, 30]. Indeed, there was no statistically significant difference in the aneuploidy rate (using FISH probes for chromosomes x, y and 18) when comparing spermatozoa with large vacuoles and vacuole-free spermatozoa (2.2% vs. 1.1%, respectively) [15] or in terms of structural chromosome aberrations in mouse oocytes injected with each type of spermatozoa (9.1% for spermatozoa with large vacuoles vs. 4.1% for vacuole-free spermatozoa) [30]. Hence, in patients with a normal karyotype, a strong link between aneuploidy and the presence of vacuoles has not been established.

Two studies have focused on patients with a high proportion of chromosomally unbalanced spermatozoa in whole semen (i.e. patients with reciprocal or Robertsonian translocations) [34, 35]. Cassuto et al. used the classification mentioned above to select different types of spermatozoa for patients with reciprocal translocations (n=6) or Robertsonian translocations (n=3) [34]. The researchers did not observe a statistically significant difference in the proportion of unbalanced segregation modes when comparing the different types of selected spermatozoa. This meant that selection of spermatozoa with a normal, vacuole-free head did not guarantee normal chromosome content. Other researchers recently reported concordant data [35]; the selection of normal spermatozoa by MSOME was no more efficient than ICSI for selecting euploid spermatozoa or excluding aneuploid spermatozoa, since the balanced translocation rates did not differ significantly (p=0.81) when comparing normal spermatozoa selected under ICSI-like magnification (56.3%) or after MSOME (53.7%) [35]. It should be noted that, in these two latter studies, the small sample size reduced the statistical significance. Hence, it is still not clear whether vacuoles are related to abnormal chromosome content in patients with a normal karyotype or patients with chromosome structure abnormalities.

#### The acrosome and sperm head vacuoles

Only one study has compared acrosome shape in spermatozoa with large vacuoles and in unselected spermatozoa in whole sperm. The researchers reported that the proportion of spermatozoa with a morphologically abnormal acrosome was significantly higher in spermatozoa with large vacuoles than in unselected spermatozoa (77.6% vs. 70.6%, respectively, p=0.014) [19].

Even though large vacuoles may be associated with acrosome morphological abnormalities, other researchers have gathered data that argue against the acrosomal nature of vacuoles. For example, vacuoles were observed in globozoospermic spermatozoa (which lack the acrosome) from two patients [36]. Furthermore, no difference in acrosome reaction status (i.e. reacted or not, as assessed by *Pisum sativum* agglutinin lectin staining) was observed when comparing morphometrically normal, vacuole-free spermatozoa with those containing large vacuoles (>25% of the sperm head area) in a study of 15 patients [15]. In fact, none of the 100 morphometrically normal spermatozoa observed (whether presenting one large vacuole or none) were acrosome-reacted [15]. More recently, a study of 15 patients found that the proportion of acrosome-reacted spermatozoa (as assessed by *Pisum sativum* agglutinin lectin staining) of individually selected morphometrically normal spermatozoa with more than 2 small vacuoles (each occupying ≤ 4% of the sperm head area) did not differ significantly from that of similar but vacuole-free spermatozoa (6.0% vs. 2.9%, respectively; p=0.15) [14].

On the other hand, it was reported that induction of the acrosome reaction with either the calcium ionophore A23587 [37], follicular fluid or hyaluronic acid [38] may be associated with a decrease in the proportion of spermatozoa with vacuoles. In a study of 10 patients, induction of the acrosome reaction was associated with an higher proportion of spermatozoa with a vacuole-free head (41.2% before induction vs. 63.8% after induction, p<0.005) [37]. Likewise, in a study of 35 patients, induction of the acrosome reaction with hyaluronic acid was associated with a lower proportion of spermatozoa containing vacuoles (44.1% before induction vs. 31.9% after induction; p<0.03) [38]. The latter researchers then suggested that the vacuoles were of acrosomal origin. However, very recently, Neyer et al. published data that disagreed with those of the two latter studies [39]. By observing the same live spermatozoa for 24 hours, they found that induction of the acrosome reaction with ionophore A23587 neither changed the proportion of spermatozoa with vacuoles nor prompted the disappearance of pre-existing vacuoles [39].

In conclusion, most of the data gathered to date indicate that vacuoles are nuclear in nature and that these vacuoles are associated with chromatin condensation failure and a potential increase in susceptibility to DNA damage. There are also many arguments in favour of a non-acrosomal nature. Hence, a decade after the first description of MSOME in the 2000s, this non-invasive technique enables (i) the visualization of nuclear structures that are associated with nuclear chromatin condensation failure and (ii) the selection of spermatozoa with the highest nuclear chromatin quality.
